# Genetic variants in *PPP2CA* are associated with gastric cancer risk in a Chinese population

**DOI:** 10.1038/s41598-017-12040-z

**Published:** 2017-09-13

**Authors:** Tongtong Huang, Kexin He, Yingying Mao, Meng Zhu, Caiwang Yan, Fei Yu, Qi Qi, Tianpei Wang, Yan Wang, Jiangbo Du, Li Liu

**Affiliations:** 1 Department of Epidemiology and Biostatistics, School of Public Health, Nanjing Medical University, Nanjing, China; 2 Jiangsu Key Lab of Cancer Biomarkers, Prevention and Treatment, Collaborative Innovation Center for Cancer Medicine, Nanjing Medical University, Nanjing, China; 3 Digestive Endoscopy Center, The First Affiliated Hospital of Nanjing Medical University and Jiangsu Province Hospital, Nanjing, China; 4 Department of Epidemiology and Biostatistics, School of Basic Medical Sciences, Zhejiang Chinese Medical University, Hangzhou, China

## Abstract

Protein phosphatase 2A (PP2A), a tumor suppressor protein, has been implicated in cell cycle and apoptosis. Additionally, studies have illustrated its crucial roles in transformation of normal human cells to tumorigenic status. *PPP2CA*, which encodes the alpha isoform of the catalytic subunit of PP2A, has been recently reported to be associated with several types of cancers. Therefore, we hypothesized that genetic variants in *PPP2CA* might influence susceptibility of gastric cancer. To test this hypothesis, three tagging single nucleotide polymorphisms (SNPs) in *PPP2CA* were genotyped in a case-control study including 1,113 cases and 1,848 controls in a Chinese population. Three tagging SNPs in *PPP2CA* were genotyped using Illumina Human Exome BeadChip. We observed that the A allele of rs13187105 was associated with an increased risk of gastric cancer (adjusted odds ratio (OR) = 1.14, 95% confidence interval (CI): 1.02–1.28, *P* = 0.017). Further analyses showed that rs13187105 [A] was associated with decreased expression of *PPP2CA* mRNA (*P* = 5.1 × 10^−6^), and *PPP2CA* mRNA was significantly lower in gastric tumor tissues when comparing that in their adjacent normal tissues (*P* = 0.037). These findings support our hypothesis that genetic variants in *PPP2CA* may be implicated in gastric cancer susceptibility in Chinese population.

## Introduction

Gastric cancer is a major public health problem around the world, which led to 951,000 incident cases and 721,000 deaths worldwide in 2012^1^. In China, the problem is more serious, with estimates of 679,100 new cases and 498,000 deaths in 2015^2^. Various risk factors are involved in gastric carcinogenesis, for instance, *Helicobacter pylori* infection, nitrites consumption and processed meat intake have been associated with risk of gastric cancer^3–8^. Besides these environmental and life style factors, host genetic factors may also play vital roles in the process of gastric cancer development, so that some individuals are prone to develop gastric cancer than the others^9^. Recently, genome-wide association studies (GWASs) have shown huge advantages in uncovering germline common variants associated with malignant tumors. Multiple susceptibility loci of gastric cancer, such as 1q22, 5p13.1, 3q13.31 and 10q23, have been identified in previous GWASs^10–12^. However, the findings of GWASs could only account for a fringe of genetic predisposition of gastric cancer because of the strict selection criteria and the limited number of single nucleotide polymorphisms (SNPs) in the arrays. Therefore, candidate gene strategy is still an important method to identify susceptibility loci in vital genes involved in carcinogenesis^13^.

Protein phosphatase 2A (PP2A), a heterotrimeric holoenzyme complex, is one of the major serine/threonine phosphatases and participates in a large proportion of phosphatase activity in eukaryotic cells^14–16^. It has been demonstrated in previous studies that the activation of telomerase and Ras, along with the inactivation of tumor suppressor proteins p53 and retinoblastoma protein are sufficient to immortalize the majority of human cells. However, these immortalized cells can hardly transform to tumorigenic status without inhibiting PP2A activity^17–20^. The PP2A core enzyme, which consists of a 36 kDa C catalytic subunit and a 65 kDa A scaffold subunit, can combine with a regulatory B subunit. There are various kinds of B subunits and different B subunits play different cellular function^21^. The C catalytic subunit of PP2A has two subtypes, α and β, and the α isoform of the catalytic subunit (PPP2Cα) is encoded by the gene *PPP2CA*. Previous studies have demonstrated that PPP2Cα plays important roles in regulating PP2A activity through mediating the selection of PP2A regulatory subunits^21–23^. *PPP2CA* has been reported to be associated with several types of cancer, including prostate cancer, non-small cell lung cancer and acute myeloid leukemia^24–26^. Bhardwaj A *et al*. observed the attenuate of migration and invasion potential of prostate cancer cells after they overexpressed *PPP2CA* ^27^. Moreover, they found that overexpression of *PPP2CA* could inhibit prostate tumor growth and metastasis in an orthotopic mouse model^27^.

However, studies of the association between genetic variants in *PPP2CA* and risk of gastric cancer are lacking. In the present study, we conducted a case-control study of 1,113 patients with gastric cancer and 1,848 cancer-free controls in a Chinese population to evaluate the associations of three tagging SNPs in *PPP2CA* with risk of gastric cancer. Furthermore, we used public databases to compare mRNA level of *PPP2CA* in gastric tumor tissues with that in adjacent normal tissues, and to explore potential function of the SNPs on gastric cancer development.

## Results

The characteristics of patients with gastric cancer and cancer-free controls included in the present study were summarized in Supplementary Table S1. Age, sex and drinking status were comparable between the cases and the controls. However, there were more smokers in the control group than in cases (52.07% vs 48.71%). In addition, the results of multivariate logistic regression analysis for age, sex, smoking and drinking status were also shown in Supplementary Table S1. The detailed information of the 3 tag SNPs genotyped was listed in Supplementary Table S2. The call rates for all three variants were 100%. Besides, they were all in Hardy-Weinberg equilibrium (HWE) among controls (*P* = 0.637 for rs13187105, *P* = 0.769 for rs2292283 and *P* = 0.819 for rs254057).

The genotype distributions of the 3 tag SNPs and their associations with risk of gastric cancer were summarized in Table 1. Logistic regression analyses showed that the A allele of rs13187105 was significantly associated with an increased risk of gastric cancer under the additive model (adjusted odds ratio (OR) = 1.14, 95% confidence interval (CI): 1.02–1.28, *P* = 0.017). However, no statistically significant association was observed for rs2292283 and rs254057 under the additive models. To further explore the association between rs13187105 and risk of gastric cancer, we performed stratified analyses by age, sex, smoking status, drinki﻿ng﻿ status and tumor site. As shown in Table 2, the association between rs13187105 and gastric cancer remained significant in the subgroups of participants ≥60 years old, men and smokers. Significant heterogeneity was observed between smokers and non-smokers (*P* for heterogeneity = 0.040). The minor allele [A] of rs13187105 was associated with an increased risk of gastric cancer among smokers (adjusted per-allele OR = 1.29, 95% CI: 1.09–1.51, *P* = 0.002), but no significant association was observed in non-smokers (adjusted per-allele OR = 1.01, 95% CI: 0.86–1.19, *P* = 0.873).

**Table 1:** Genotype frequencies of the 3 tag SNPs in *PPP2CA* and their associations with gastric cancer risk.

genotypes	Cases(n = 1,113)		Controls(n = 1,848)		OR(95% CI)^a^	*P* value^a^
	N	%	N	%		
rs13187105						
CC	324	29.11	572	30.95	1.00	
CA	515	46.27	922	49.89	0.97(0.81–1.16)	0.708
AA	274	24.62	354	19.16	1.34(1.08–1.67)	0.008
Additive model					1.14(1.02–1.28)	0.017
rs2292283						
GG	399	35.85	692	37.45	1.00	
GA	501	45.01	883	47.78	0.95(0.80–1.13)	0.599
AA	213	19.14	273	14.77	1.30(1.04–1.64)	0.024
Additive model					1.10(0.99–1.23)	0.080
rs254057						
GG	981	88.14	1653	89.45	1.00	
GA	127	11.41	189	10.23	1.20(0.93–1.54)	0.158
AA	5	0.45	6	0.32	1.38(0.40–4.77)	0.607
Additive model					1.20(0.95–1.51)	0.136

^a^Adjusted for age, sex, smoking status, drinking status and the top ten principal components.

**Table 2:** Stratified analyses of associations between rs13187105 and gastric cancer risk.

Variables	Case	Control	OR(95% CI)^a^	*P* ^a^	*P* ^b^
	CC/CA/AA	CC/CA/AA			
Age (years)					
<60	139/226/101	250/416/161	1.07(0.90–1.27)	0.441	0.354
≥60	185/289/173	322/506/193	1.19(1.03–1.38)	0.021	
Sex					
Male	246/387/206	424/673/246	1.17(1.03–1.34)	0.016	0.378
Female	78/128/68	148/249/108	1.04(0.83–1.31)	0.717	
Smoking status					
Never	176/267/135	255/439/180	1.01(0.86–1.19)	0.873	0.040
Ever	147/248/139	317/483/174	1.29(1.09–1.51)	0.002	
Drinking status					
Never	191/314/158	344/551/220	1.12(0.97–1.29)	0.125	0.752
Ever	133/201/116	228/371/134	1.16(0.97–1.39)	0.100	
Tumor site					
Cardia	163/243/140	572/922/354	1.15(1.00–1.32)	0.056	0.911
Non-cardia	161/272/134	572/922/354	1.14(0.99–1.30)	0.074	

^a^Derived from additive models using logistic regression analyses with adjustments for age, sex, smoking status, drinking status and the top ten principal components. ^b^ *P* for heterogeneity test based on χ2-based Q test.

**Figure 1:**
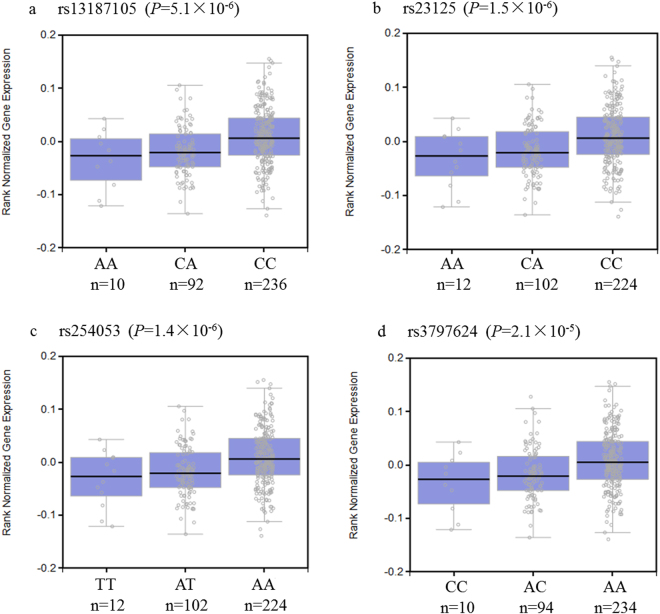
Expression quantitative trait loci (eQTL) analyses of rs13187105 (**a**), rs23125 (**b**), rs254053 (**c**), rs3797624 (**d**) with *PPP2CA* mRNA expression levels in the whole blood samples. The results indicated that minor alleles of rs13187105, rs23125, rs254053 and rs3797624 were significantly correlated with decreased expression levels of *PPP2CA*. The *p* values were derived from linear regression model. The data was obtained from Genotype-Tissue Expression project (GTEx V6p) Portal.

**Figure 2:**
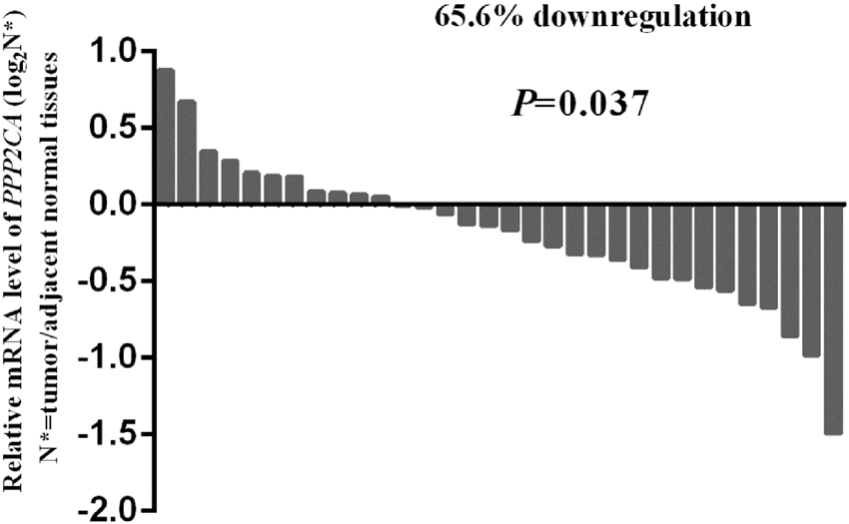
*PPP2CA* mRNA expression levels in 32 paired gastric tumor and adjacent normal tissues. Compared with adjacent normal controls, the expression level of *PPP2CA* was decreased in tumor tissues. The *p* value was derived from paired Student’s t-test. The data was obtained from The Cancer Genome Atlas (TCGA) database.

Based on Genotype-Tissue Expression project (GTEx) portal, we compared the expression ﻿of *PPP2CA* among individuals with different genotypes and found that the risk allele of rs13187105 was associated with reduced expression of *PPP2CA* (*P* = 5.1 × 10^−6^) in peripheral blood samples (Fig. 1a)^28^. Moreover, using The Cancer Genome Atlas (TCGA) database, we found that the mRNA level of *PPP2CA* was decreased in gastric cancer tissues compared to the adjacent normal tissues (*P* = 0.037) (Fig. 2)^29^. These findings suggested that *PPP2CA* is a potential suppressor gene of gastric cancer, and its expression might be regulated by rs13187105 or some other SNPs in strong linkage disequilibrium (LD) with rs13187105. Therefore, using HaploReg v4.1 database, we subsequently analyzed 81 SNPs in strong LD (r^2^ > 0.8) with rs13187105 to explore the potential causal SNPs with real biological function. We found that 12 SNPs are located in the promoter region, and 5 of them (rs254051, rs2284317, rs23125, rs254053, rs3797624) are predicted to be bound with proteins (Supplementary Table S3). We also annotated these SNPs based on UCSC genome browser, and the findings were shown in Supplementary Fig. S1. Briefly, according to the Encyclopedia of DNA Elements (ENCODE) data, 3 SNPs (rs23125, rs254053 and rs3797624) are located in the peak of H3K4me3 chromatin modification (7 cell lines) and the region is also enriched with DNase Hypersensitivity clusters and transcription factor ChIP-seq signals. Data from the Roadmap Epigenomics also reveals that these 3 SNPs are located in the peak of H3K4me3 chromatin modification and candidate active transcription start site in stomach mucosa. In addition, results from GTEx portal showed that the minor alleles of all these 3 SNPs were associated with reduced *PPP2CA* expression (*P* = 1.5 × 10^−6^ for rs23125, *P* = 1.4 × 10^−6^ for rs254053 and *P* = 2.1 × 10^−5^ for rs3797624) (Fig. 1b–d). These results provided evidence that the 3 potential causal SNPs (rs23125, rs254053 and rs3797624), in complete LD with rs13187105 (Supplementary Table S3), are located in the functional promoter region of *PPP2CA* gene and may regulate the expression of *PPP2CA*.

## Discussion

In this study, we genotyped 3 tag SNPs in *PPP2CA* and evaluated their associations with risk of gastric cancer in a case-control study including 1,113 cases and 1,848 controls in a Chinese population. We found that rs13187105 in *PPP2CA* was significantly associated with an altered risk of gastric cancer.

In addition, *in silico* analyses showed that the minor allele [A] of rs13187105 was significantly associated with reduced *PPP2CA* mRNA expression in peripheral blood samples. Moreover, *PPP2CA* expression was lower in gastric tumor tissues compared to that in adjacent normal tissues. Further analyses indicated that 3 potential causal SNPs (rs23125, rs254053and rs3797624) in complete LD with rs13187105 are located in the functional promoter region of *PPP2CA* gene and may regulate the expression of *PPP2CA*. We also evaluated the associations between the 4 SNPs (rs13187105, rs23125, rs254053 and rs3797624) and the expression of *PPP2CA* in gastric mucosa samples, and the data was shown in Supplementary Fig. S2 ^28^. Though the *p* values did not reach statistical significance (*P* < 0.05), the directions of the associations were consistent when comparing with that in whole blood samples. The possible reason is that the sample size of gastric mucosa (n = 170) is much smaller than that of whole blood (n = 338).

In Table 1, We also found that rs2292283 AA genotype was associated with the risk of gastric cancer compared with GG genotype (adjusted OR = 1.14, 95% CI: 1.04–1.64, *P* = 0.024). The association between rs2292283 AA genotype and risk of gastric cancer might due to the strong LD between rs2292283 and rs13187105 (r^2^ = 0.71).

In our study, after Bonferroni correction, the *p* value of rs13187105 in additive model was 0.051, which was marginal associated with risk of gastric cancer. We also used recessive model to access the association between rs13187105 and the risk of gastric cancer, and we found that the AA genotype of rs13187105 was significantly associated with an increased risk of gastric cancer compared with CC+CA genotypes (adjusted odds ratio (OR) = 1.37, 95% confidence interval (CI): 1.14–1.66, *P* = 0.001), and after Bonferroni correction, the *p* value of rs13187105 under recessive model was 0.003.

PP2A is a bona fide tumor suppressor protein which plays essential roles in the regulation of cell cycle, apoptosis, transcription and DNA repair. PP2A holoenzymes are estimated to be responsible for 30%–50% of total cellular serine/threonine dephosphorylating activity^30–32^. In addition, PP2A is an important protein in malignant transformation. *PPP2CA* encodes the alpha isoform of the catalytic subunit of PP2A, and its methylation and phosphorylation play essential roles in the selection of PP2A regulatory B subunits, which are important for PP2A activity. Previous study indicated that the activity of PP2A was downregulated after decreasing the expression level of *PPP2CA* ^33^. Therefore, it is biological plausible that decreased expression level of *PPP2CA* might affect PP2A activity in stomach tissues and then further influence the process of carcinogenesis.

According to our findings, the risk allele of rs13187105 was significantly associated with the reduced expression of *PPP2CA*, and the 3 candidate casual SNPs (rs23125, rs254053 and rs3797624) in complete LD with rs13187105 are located within the putative promoter regulatory regions. Therefore, the decreased expression of *PPP2CA* might be partially responsible for the observed association between rs13187105 and the risk of gastric cancer.

Our study was the first to explore the associations between genetic variants in *PPP2CA* and risk of gastric cancer. Some potential limitations of our study merit discussion. First, there were more smokers in the control group than in cases (52.07% vs 48.71%). The smoking status of gastric patients was collected in hospitals and some patients had changed their lifestyle since they were diagnosed with gastric cancer or they were likely to falsify their smoking history when information collection, as a result, bias might exit in our case-control study. To control the influence of bias, smoking status was adjusted when calculated the associations between SNPs and gastric cancer risk. Therefore, the bias might exert less impact to the results of the associations. Second, we did not have access to other independent study populations to confirm our findings. However, the sample size of the current study was relatively large, which at some level ensure the study quality. What’s more, the annotations of the SNPs were all based on public databases. Further functional studies are warranted to clarify the underlying biological mechanisms.

Taken together, our study found that the minor allele [A] of rs13187105 was associated with an increased risk of gastric cancer and meanwhile associated with decreased *PPP2CA* expression. These findings might stimulate further research investigating the roles of *PPP2CA* in the development of gastric cancer.

## Materials and Methods

### Study subjects

The details of the study participants including 1,113 gastric cancer cases and 1,848 cancer-free controls had been described previously^34^. Briefly, the cases were histopathologically or cytologically confirmed primary gastric carcinoma, and were recruited from the hospitals in Jiangsu province, China between January 2004 and December 2011. Those with previous history of malignancies or had undergone radiotherapy or chemotherapy were excluded. The control subjects were randomly selected from healthy individuals in a community-based screening program for chronic non-communicable diseases conducted in Jiangsu province and were frequency matched to cases by age and sex. All subjects were unrelated Chinese Han populations. After written informed consent was obtained, each participant was interviewed to collect demographic information and related risk factors of gastric cancer. After the interview, peripheral blood sample was collected from each subject. Individuals who smoked at least once per day for more than one year were classified as smokes, and those who drank more than twice a week for no less than a year were considered as drinkers. The study was approved by the institutional review board of Nanjing Medical University and all procedures were performed in accordance with relevant guidelines and regulations. Written informed consent was obtained from every participant.

### Selection of tag SNPs and genotyping

The HapMap database (HapMap Data Rel 27 phase II+III, Feb09) was used to retrieve SNP information in *PPP2CA* (including 10kb upstream region) in the Chinese Han population (CHB). Haploview 4.2 software was used to select tag SNPs with r^2^ threshold of 0.80 and minor allele frequency (MAF) ≥ 0.05. Finally, 3 SNPs including rs13187105 (C > A), rs2292283 (G > A) and rs254057 (G > A) were selected for further analyses.

Genomic DNA was extracted from peripheral leukocytes using phenol-chloroform method. The tag SNPs were genotyped in all participants using Illumina Human Exome BeadChip, in which these three loci were customer-designed. Genotype calling was done using the Illumina GenomeStudio software.

### Public database searching

The HapMap database (http://hapmap.ncbi.nlm.nih.gov/cgi-perl/gbrowse/hapmap27_B36/) was used to download the genotype information of *PPP2CA* in the CHB population. The Expectation-Maximization (RSEM) normalized read counts of *PPP2CA* in 32 paired gastric tumors and adjacent normal tissues were downloaded from the TCGA database (https://cancergenome.nih.gov/, accessed on April 8, 2015). The GTEx V6p Portal (http://www.gtexportal.org/home/) was used to perform the expression quantitative trait loci (eQTL) analyses in the whole blood samples (n = 338) and in the stomach mucosa samples (n = 170). The LD information of rs13187105 was calculated based on the data from 1000 Genomes project (phase3) (https://www.ncbi.nlm.nih.gov/variation/tools/1000genomes/) in CHB population. Functional annotations were performed using the HaploReg v4.1 database (http://archive.broadinstitute.org/mammals/haploreg/haploreg.php) and UCSC genome browser (https://genome.ucsc.edu/cgi-bin/hgGateway).

### Statistical analysis

Departures from HWE were tested by the goodness-of-fit χ2 test to compare the observed genotype frequencies to the expected ones among the control subjects. Differences in the distribution of categorical variables including age, sex, smoking and drinking status were evaluated by χ^2^ test and age was compared by Welch’s t-test when regarded as a continuous variable. Multivariate logistic regression analysis for age, sex, smoking and drinking status was also done to assess their effects on gastric cancer susceptibility. To evaluate the associations between the tag SNPs and gastric cancer risk, unconditional logistic regression models were used to calculate ORs and their 95% CIs adjusted for age, sex, smoking status, drinking status and the top ten principal components. The detailed information about the top ten principal components could be obtained from our previous study^34^. In brief, the population structure was evaluated using principal component analysis using EIGENSOFT4.2 based on 4,861 autosomal scaffold markers included in the exome array. No population outliers were detected, suggesting that the study subjects were genetically matched. Therefore, to avoid the effect of the population structure, the top ten principal components were included in the logistic regression model as covariates. The *p* values of eQTL were obtained from linear regression model. Paired Student’s t-test was used to compare the *PPP2CA* expression levels of 32 gastric tumor and adjacent normal tissue pairs. All analyses were performed using R3.2.3 and plink1.07. Two-sided *P*-values less than 0.05 were considered as statistically significant.

### Data Availability

The datasets generated and analyzed during the current study are available from the corresponding author on reasonable request.

## Electronic supplementary material

supplementary infomation
