# Supplementary material for: Genetic variants in PPP2CA are associated with gastric cancer risk in a Chinese population
Source: Sci Rep. 2017 Sep 13;7:11499. doi: 10.1038/s41598-017-12040-z (PMC5597632; doi:10.1038/s41598-017-12040-z)
Supplement: Supplementary file 1 — supplementary infomation [file 41598_2017_12040_MOESM1_ESM.pdf]

Genetic variants in *PPP2CA* are associated with gastric cancer risk in a Chinese population

Tongtong Huang[1,2†], Kexin He[3†], Yingying Mao[1,4], Meng Zhu[1], Caiwang Yan[1], Fei Yu[1], Qi Qi[1], Tianpei Wang[1], Yan Wang[3], Jiangbo Du[1,2*], Li Liu[3*]

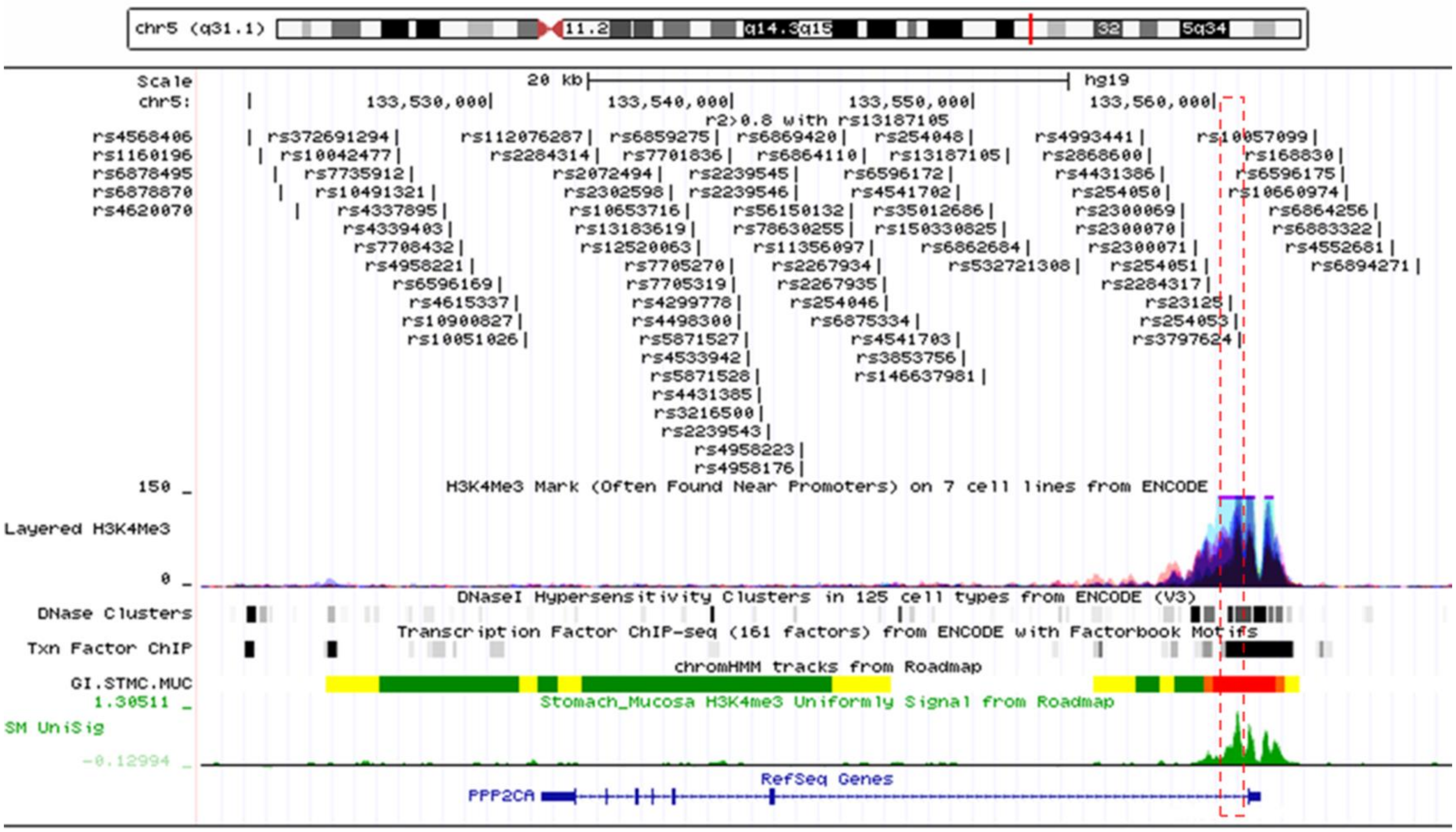

Supplementary Figure S1. The functional annotations of the 80 SNPs showing strong linkage disequilibrium (r^2>0.8) with rs13187105 in UCSC genome browser. SNPs in the red dotted frame (rs23125, rs254053, rs3797624) are located within the putative promoter transcription regulatory region characterized by H3k4me3 histone protein modification, DNase Hypersensitivity clusters and transcription factor-binding sites. Red segmentation area in the chromHMM track of stomach mucosa is annotated as candidate active transcription start site.

*****rs5871539 is not shown in the figure because it is far away from others.

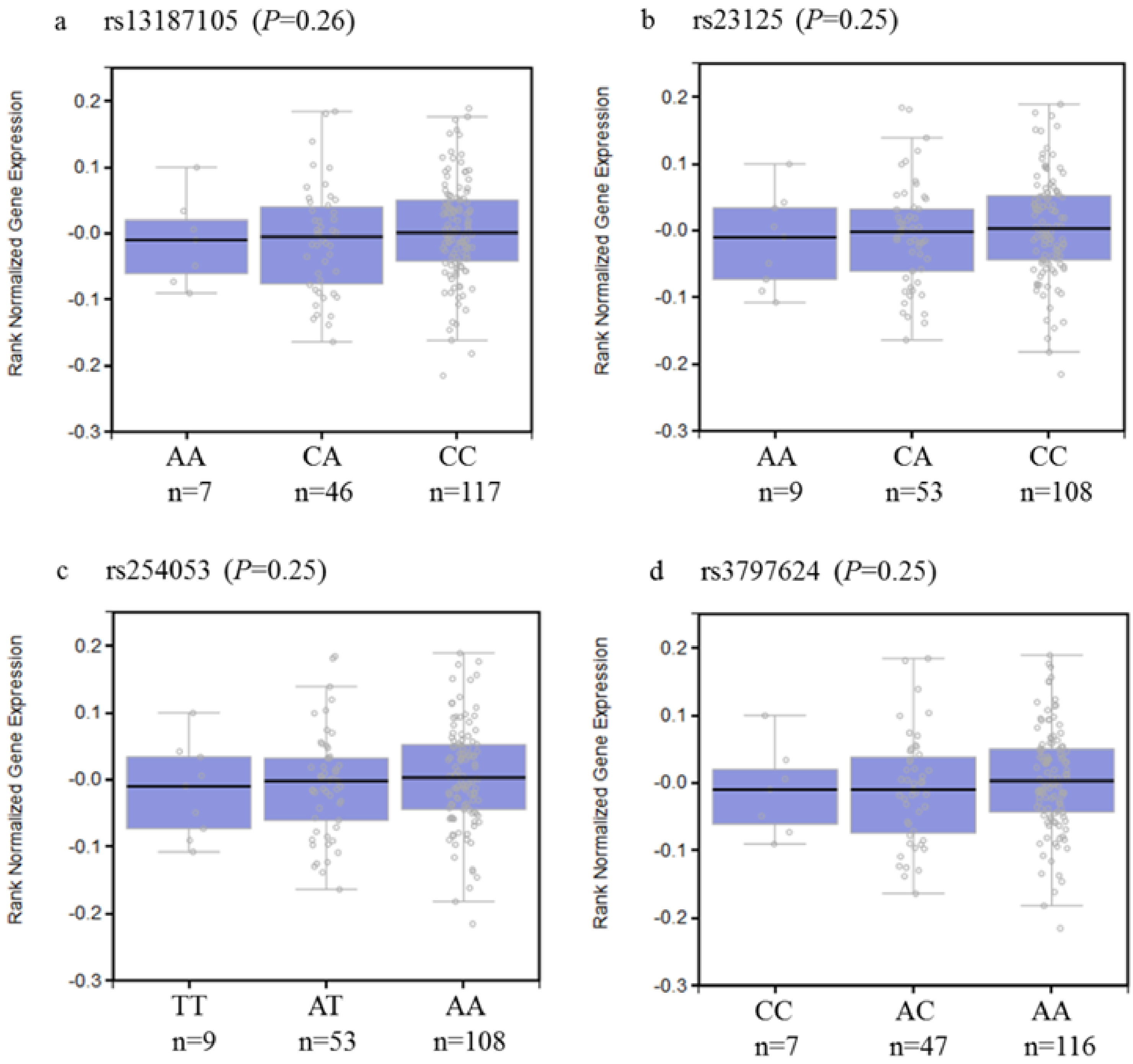

Supplementary Figure S2. Expression quantitative trait loci (eQTL) analyses of rs13187105 (a), rs23125 (b), rs254053 (c), rs3797624 (d) with *PPP2CA* mRNA expression levels in the gastric mucosa samples. The directions of the associations were consistent with the ones in the whole blood samples. The *p* values were derived from linear regression model.The data was obtained from Genotype-Tissue Expression project (GTEx V6p) Portal.

Supplementary Table S1. Demographic characteristics of the subjects in this study.

Variable	Case (n=1,113)	Control (n=1,848)	OR [a]	*P* [a]	*P*
	N (%)	N (%)			
Age (mean ± SD)	61.02 ± 10.51	60.76 ± 12.05			0.544 [b]
< 60	466(41.87)	827(44.75)	1.00	0.161	0.126 [c]
≥ 60	647(58.13)	1021(55.25)	1.11		
Sex					
Male	839(75.38)	1343(72.67)	1.00	0.002	0.105 [c]
Female	274(24.62)	505(27.33)	0.73		
Smoking status					
Never	578(51.93)	874(47.29)	1.00	<0.001	0.014 [c]
Ever	535(48.07)	974(52.71)	0.71		
Drinking status					
Never	663(59.57)	1115(60.34)	1.00	0.360	0.680 [c]
Ever	450(40.43)	733(39.67)	1.08		
Tumor site					
Cardia	546(49.06)				
Non-cardia	567(50.94)				

[a] Logistic regression analyses with adjustments for age, sex, smoking and drinking status (except the variable being analyzed).

[b] Welch's t-test was applied for the unequal variances.

[c] Two-sided $\chi 2$ test was used.

Supplementary Table S2. Detailed information of 3 tag SNPs in *PPP2CA*.

Gene	SNP	position(hg19)	Location	Major/Minor	Call Rate	HWE(control)	MAF	MAF(case/control)
PPP2CA	rs13187105	chr5:133551445	intronic	C/A	100%	0.637	0.455	0.478/0.441
PPP2CA	rs2292283	chr5:133563318	nearGene-5'	G/A	100%	0.769	0.398	0.416/0.387
PPP2CA	rs254057	chr5:133571856	nearGene-5'	G/A	100%	0.819	0.057	0.062/0.054

SNP: single nucleotide polymorphism; HWE: Hardy-Weinberg equilibrium; MAF: minor allele frequency.

Supplymentary Table S3. The bioinformatics prediction of SNPs which showed strong linkage disequilibrium (r^2>0.8) with rs13187105.

SNP	Gene	position(hg19)	Location	$r^{2\ a}$	Promoter histone marks	proteins bound
rs4568406		chr5:133519949	intergenic	1.00		YES
rs1160196		chr5:133520447	intergenic	1.00		
rs6878495		chr5:133521086	intergenic	1.00		
rs6878870		chr5:133521309	intergenic	1.00		
rs4620070		chr5:133521953	intergenic	1.00		
rs372691294[b]		chr5:133526116	intergenic	0.81	—	—
rs10042477		chr5:133526144	intergenic	0.98		
rs7735912		chr5:133526689	intergenic	0.98		YES
rs10491321		chr5:133527590	intergenic	0.98		
rs4337895		chr5:133528107	intergenic	0.98		
rs4339403		chr5:133528322	intergenic	0.98		
rs7708432		chr5:133528730	intergenic	0.98		
rs4958221		chr5:133529191	intergenic	0.98		
rs6596169		chr5:133530369	intergenic	1.00		
rs4615337		chr5:133531028	intergenic	0.98		YES
rs10900827		chr5:133531205	intergenic	0.98		
rs10051026		chr5:133531449	intergenic	0.98		
rs112076287	*PPP2CA*	chr5:133534086	intronic	0.98		
rs2284314	*PPP2CA*	chr5:133534413	intronic	0.98		
rs2072494	*PPP2CA*	chr5:133537009	intronic	0.98		
rs2302598	*PPP2CA*	chr5:133537438	intronic	0.98		
rs10653716	*PPP2CA*	chr5:133538126	intronic	1.00		
rs13183619	*PPP2CA*	chr5:133538384	intronic	1.00		
rs12520063	*PPP2CA*	chr5:133538600	intronic	1.00		
rs6859275	*PPP2CA*	chr5:133539363	intronic	1.00		
rs7701836	*PPP2CA*	chr5:133539947	intronic	1.00		
rs7705270	*PPP2CA*	chr5:133539999	intronic	1.00		
rs7705319	*PPP2CA*	chr5:133540070	intronic	1.00		
rs4299778	*PPP2CA*	chr5:133540259	intronic	1.00		
rs4498300	*PPP2CA*	chr5:133540325	intronic	1.00		
rs5871527	*PPP2CA*	chr5:133540628	intronic	0.83		
rs4533942	*PPP2CA*	chr5:133540658	intronic	1.00		
rs5871528	*PPP2CA*	chr5:133541079	intronic	0.83		
rs4431385	*PPP2CA*	chr5:133541159	intronic	1.00		
rs3216500	*PPP2CA*	chr5:133541239	intronic	0.98		
rs2239543	*PPP2CA*	chr5:133541548	intronic	1.00		
rs2239545	*PPP2CA*	chr5:133542659	intronic	1.00		
rs2239546	*PPP2CA*	chr5:133542666	intronic	1.00		

rs4958223	*PPP2CA*	chr5:133542865	intronic	1.00		
rs4958176	*PPP2CA*	chr5:133542915	intronic	1.00		
rs6869420	*PPP2CA*	chr5:133544636	intronic	1.00		
rs56150132	*PPP2CA*	chr5:133544970	intronic	0.82		
rs78630255	*PPP2CA*	chr5:133544972	intronic	0.82		
rs6864110	*PPP2CA*	chr5:133545491	intronic	1.00		
rs11356097	*PPP2CA*	chr5:133545800	intronic	0.9		
rs2267934	*PPP2CA*	chr5:133546135	intronic	1.00		
rs2267935	*PPP2CA*	chr5:133546309	intronic	1.00		
rs254046	*PPP2CA*	chr5:133546437	intronic	1.00		
rs6875334	*PPP2CA*	chr5:133547684	intronic	1.00		
rs6596172	*PPP2CA*	chr5:133549090	intronic	1.00		
rs4541702	*PPP2CA*	chr5:133549367	intronic	1.00		
rs4541703	*PPP2CA*	chr5:133549375	intronic	1.00		
rs3853756	*PPP2CA*	chr5:133549638	intronic	1.00		
rs254048	*PPP2CA*	chr5:133549915	intronic	1.00		
rs146637981	*PPP2CA*	chr5:133550455	intronic	1.00		
rs35012686	*PPP2CA*	chr5:133550730	intronic	0.93		
rs150330825	*PPP2CA*	chr5:133551266	intronic	1.00		
rs13187105	*PPP2CA*	chr5:133551445	intronic	1.00		
rs6862684[b]	*PPP2CA*	chr5:133552248	intronic	1.00	—	—
rs532721308[b]	*PPP2CA*	chr5:133554360	intronic	0.93	—	—
rs4993441	*PPP2CA*	chr5:133557039	intronic	1.00	YES	
rs2868600	*PPP2CA*	chr5:133557316	intronic	1.00	YES	
rs4431386	*PPP2CA*	chr5:133557876	intronic	1.00	YES	
rs254050	*PPP2CA*	chr5:133558054	intronic	1.00	YES	
rs2300069	*PPP2CA*	chr5:133558683	intronic	1.00	YES	
rs2300070	*PPP2CA*	chr5:133558692	intronic	1.00	YES	
rs2300071	*PPP2CA*	chr5:133559214	intronic	1.00	YES	
rs254051	*PPP2CA*	chr5:133559697	intronic	1.00	YES	YES
rs2284317	*PPP2CA*	chr5:133559757	intronic	1.00	YES	YES
**rs23125**	***PPP2CA***	**chr5:133560646**	**intronic**	**1.00**	**YES**	**YES**
**rs254053**	***PPP2CA***	**chr5:133560931**	**intronic**	**1.00**	**YES**	**YES**
**rs3797624**	***PPP2CA***	**chr5:133561061**	**intronic**	**1.00**	**YES**	**YES**
rs10057099		chr5:133564164	intergenic	1.00		
rs168830		chr5:133565269	intergenic	1.00		
rs6596175		chr5:133565335	intergenic	1.00		
rs10660974		chr5:133565485	intergenic	1.00		
rs6864256		chr5:133566705	intergenic	1.00		
rs6883322		chr5:133566905	intergenic	1.00		
rs4552681		chr5:133567441	intergenic	1.00		
rs6894271		chr5:133568505	intergenic	1.00		
rs5871539	*CDKN2AIPNL*	chr5:133742947	intronic	0.93		

SNP: single nucleotide polymorphism

[a] r^2 with rs13187105; [b] rs372691294, rs6862684 and rs532721308 were not found in HeploReg V4.1.
